# Clinical characteristics and sociodemographic features of psychotic major depression

**DOI:** 10.1186/s12991-021-00341-7

**Published:** 2021-03-26

**Authors:** Meng-qi Wang, Ran-ran Wang, Yu Hao, Wei-feng Xiong, Ling Han, Dong-dong Qiao, Juan He

**Affiliations:** 1 School of Traditional Chinese Medicine, Beijing University of Chinese Medicine, No 11, Bei San Huan East Road, Chaoyang District, Beijing, 100029 China; 2 Shandong Provincial Mental Health Hospital, No 49, Wenhua East Road, Jinan, 250014 Shandong China

## Abstract

**Background:**

Psychotic major depression (PMD) is a subtype of depression with a poor prognosis. Previous studies have failed to find many differences between patients with PMD and those with non-psychotic major depression (NMD) or schizophrenia (SZ). We compared sociodemographic factors (including season of conception) and clinical characteristics between patients with PMD, NMD, and schizophrenia. Our aim was to provide data to help inform clinical diagnoses and future etiology research.

**Methods:**

This study used data of all patients admitted to Shandong Mental Health Center from June 1, 2016 to December 31, 2017. We analyzed cases who had experienced an episode of PMD (International Classification of Diseases, Tenth Revision codes F32.3, F33.3), NMD (F32.0–2/9, F33.0–2/9), and SZ (F20–20.9). Data on sex, main discharge diagnosis, date of birth, ethnicity, family history of psychiatric diseases, marital status, age at first onset, education, allergy history, and presence of trigger events were collected. Odds ratios (OR) were calculated using logistic regression analyses. Missing values were filled using the k-nearest neighbor method.

**Results:**

PMD patients were more likely to have a family history of psychiatric diseases in their first-, second-, and third-degree relatives ([OR] 1.701, 95% confidence interval [CI] 1.019–2.804) and to have obtained a higher level of education (OR 1.451, 95% CI 1.168–1.808) compared with depression patients without psychotic features. Compared to PMD patients, schizophrenia patients had lower education (OR 0.604, 95% CI 0.492–0.741), were more often divorced (OR 3.087, 95% CI 1.168–10.096), had a younger age of onset (OR 0.934, 95% CI 0.914–0.954), less likely to have a history of allergies (OR 0.604, 95% CI 0.492–0.741), and less likely to have experienced a trigger event 1 year before first onset (OR 0.420, 95% CI 0.267–0.661). Season of conception, ethnicity, and sex did not differ significantly between PMD and NMD or schizophrenia and PMD.

**Conclusions:**

PMD patients have more similarities with NMD patients than SZ patients in terms of demographic and clinical characteristics. The differences found between PMD and SZ, and PMD and NMD correlated with specificity of the diseases. Furthermore, allergy history should be considered in future epidemiological studies of psychotic disorders.

**Supplementary Information:**

The online version contains supplementary material available at 10.1186/s12991-021-00341-7.

## Background

Psychotic major depression (PMD) is a serious mental disorder, where patients suffer from a combination of low mood and psychosis. PMD is a subtype of major depressive disorder according to the classification systems of psychiatric diseases, the DSM-V and ICD-10 [1], and accounts for about 14.7%–18.5% of patients with major depression [2, 3]. However, compared with major depression without psychotic features, PMD is associated with longer duration [4], greater morbidity and mortality [5], lower response to antidepressants and psychotherapy [3, 6], higher rate of suicide risk [6], higher comorbidity of anxiety disorders [7], cognitive dysfunction [8], somatic disorders, and personality disorders [9, 10].

Psychotic symptoms are a risk factor for conversion from unipolar depression to bipolar disorder [11, 12] or schizophrenia (SZ) [13]. A longitudinal study of patients with PMD admitted to hospital for the first time showed that 41% of patients who were initially diagnosed with PMD, met DSM-IV criteria for bipolar disorder or schizoaffective disorder within 2 years [14]. Furthermore, depression is common in different stages of SZ and may play a role in the progression of SZ, which has raised questions around the validity of a PMD diagnosis [15, 16]. Therefore, further work on the differential etiology of PMD from other psychoses is needed [17].

In general, previous studies have failed to find many demographic differences between patients with PMD and those without psychotic features [18]. However, there have been some exceptions; family history [19, 20] of psychosis increased the risk for PMD, and ethnicity [4, 14] and educational attainment [4, 19, 21] were correlated with psychotic features in depression patients.

Studies comparing PMD and SZ have found differences in sex [19, 22], age of onset [19, 22], and marital status [23]. A number of psychosocial risk factors have also been associated with a follow-up diagnosis of PMD and SZ, which include living alone, having basic-level education, being unemployed, having less than monthly contact with friends, having no close confidants, and having experienced childhood adversity [17]. Family history of psychosis has long been recognized as an important predictor of schizophrenia [24] and major depression [25]. However, whether there is a difference in the probability of family history of mental disorder between patients with schizophrenia/non-psychotic major depression (NMD) and PMD has not been investigated. Previous studies have investigated potential associations between season of birth and major depression [26, 27, 30] as well as SZ [28]. However, in most studies, PMD was either not identified as a separate condition [26, 29] or was excluded [27]. To the best of our knowledge, only a few studies have investigated the association between season of conception and psychotic features in patients with major depression.

There have been consistent reports of an association between pollen allergy symptoms and low mood among patients with seasonal mental disorder [30–32]. An epidemiological study has demonstrated that the prevalence of autoimmune disease was significantly higher in individuals with SZ and their parents compared with unaffected controls [33]. We hypothesize that a history of allergies can help identify different psychotic conditions. Thus, we also investigated allergy history in our study.

In this study, inpatients with PMD were compared with those with NMD and SZ using measures of sociodemographic factors (including season of conception) and clinical characteristics. We examined the relative importance of various clinical features of PMD to identify characteristics that are reliably associated with the diagnosis of PMD.

## Methods

### Setting

This paper was based on data from patients admitted to Shandong Mental Health Center from June 1, 2016 to December 31, 2017. Shandong Mental Health Center is the only provincial psychiatric hospital in Shandong province, with more than 900 inpatient beds. The database from which our data were acquired contained patients’ number of admissions, age, sex, admission year, main discharge diagnosis, date of birth, ethnicity, family history of psychiatric diseases, marital status, age at first onset, education, allergy history, and presence of trigger events. For each patient, the main discharge diagnosis was made by a qualified psychiatrist at time of discharge from the hospital. Diagnoses were based on the ICD-10 Classification of Mental and Behavioural Disorders: Clinical Descriptions and Diagnostic Guidelines [34].

### Sample

Inclusion criteria were: patients who had experienced an episode of PMD (International Classification of Diseases, Tenth Revision codes F32.3, F33.3), depression (F32.0–2/9, F33.0–2/9), and SZ (F20–20.9). In total, 1788 cases were identified.

Exclusion criteria were: evidence of psychotic symptoms precipitated by an organic cause or developmental retardation (10 patients with SZ, 7 with NMD, and 3 with PMD), repeated admissions of the same patient (a total of 242 multiple admissions, only the record of the first admission was included), and patients who were not born in Shandong province (to minimize the influence of provincial cultural differences, 78 patients with SZ, 27 with NMD, and 5 with PMD). The final totals of included patients were 98 patients with PMD, 351 with NMD, and 967 with SZ.

### Clinical characteristics

Family history of psychiatric diseases was defined as at least one family member with a diagnosis of mental disorder within the patient’s first-, second-, or third-degree relatives. Age of onset was defined as the patient’s age at the first episode of the disorder. Trigger events were defined as stressful life events [35] (e.g., changing jobs, death in the family, failing exams, relationship problems, and other events that induced negative emotions in the patient) reported by the patient or their accompanying family members during the year prior to their first diagnosis. A positive allergy history was defined as having an allergy to food, drugs, pollen, etc.

### Sociodemographic factors

Marital status was determined by the patient's relationship status at the time of treatment. For example, if a patient had been divorced but had remarried at the time of the medical record, they were classified as married. Education was divided into four categories: primary school or below, junior middle school (compulsory education, usually between the ages of 12 and 15 years) or vocational school (students who do not meet the high school entrance exam score go to vocational school to learn a professional skill), senior high school or junior college (students who do not meet the university entrance exam score go to junior college), and university or higher. The Chinese population is made up of 56 distinctive ethnic groups, with Han Chinese comprising 92% of the population. Therefore, ethnicity was classified as either Chinese Han or one of the other 55 ethnic minorities.

Date of conception was calculated based on patients’ date of birth. Expected delivery date is calculated as 280 days from the first day of the last menstrual period of a pregnant woman. We assumed that fertilization occurred during ovulation (i.e., the 14th day of the last menstrual period), which would indicate that the date of birth minus 266 days would be the date of conception. The season of conception was classified according to the month of conception (spring: February–April; summer: May–August, autumn: September–October, and winter: November–January). All factors were self-reported or reported by family members who accompanied the patient.

### Statistical analyses

The data were analyzed using SPSS 22.0 and R 3.6.1 for Windows (64 bit). Data are presented as number and percentages for categorical variables, and non-normal continuous variables are reported as medians (interquartile range [IQR]). Mann–Whitney *U* tests were used to compare age of onset between NMD and PMD and between PMD and schizophrenia. Differences in missing data between diagnostic groups were compared using Fisher’s exact test. Intergroup differences were compared using a Mantel Haenszel Chi-square test or Fisher’s exact test for categorical variables. Variables with *p* < 0.10 were included in the logistic regression analysis. Family history of psychiatric diseases and sex were included in the model as effect-modifying variables. Odds ratios (OR) were calculated using logistic regression analyses. Missing values were filled using the K-nearest neighbor method (*k* = 15 and meth = median, so that the most frequent value of the 15 neighbors were filled in the missing values) [36]. The findings were considered statistically significant when the two-tailed analyses resulted in *p* < 0.05. Categorical variables were transformed into serial numbers: sex (1 = male, 2 = female), marital status (1 = unmarried, 2 = married, 3 = widowed, 4 = divorced), family history of psychiatric illness (1 = yes, 0 = no), allergy history (1 = yes, 0 = no), season of conception (1 = spring, 2 = summer, 3 = autumn, 4 = winter), education (1 = primary school or below, 2 = junior high or vocational school, 3 = senior high school or junior college, 4 = university and above), and ethnicity (1 = Han, 2 = minority).

## Results

### Distribution of sociodemographic factors and clinical characteristics

The distribution of sociodemographic factors and clinical characteristics is presented in Table 1. Patients with PMD were significantly more educated than patients with NMD and SZ (*χ*^2^ = 11.631, *p* < 0.001 for NMD and *χ*^2^ = 40.888, *P* < 0.001 for SZ). Patients with SZ were significantly younger at the first psychotic episode (*U* = 31,244.50, *p* < 0.001) than those with PMD. No statistically significant differences were observed between the PMD and NMD groups’ age of onset (*U* = 15,969.5, *p* = 0.317). The percentage of unmarried patients in the PMD group was significantly lower than that in the SZ group (*χ*^2^ = 16.253, *p* < 0.001), but was not significantly different to the NMD group (*χ*^2^ = 2.118, *p* = 0.555). Patients with SZ were significantly less likely to have a history of allergy compared with patients with PMD (*χ*^2^ = 7.545, *p* = 0.006). There was no significant difference in allergy history between the PMD and NMD groups (*χ*^2^ = 2.463, *p* = 0.155).

**Table 1:** Distribution of social–demographic factors and clinical characteristics

	NMD *n* (%)	PMD *n* (%)	SZ *n* (%)
Marital status			
Unmarried	93 (27)	33 (33.7)	474 (49.4)*
Married	222 (64.5)	58 (59.2)	368 (38.4)
Widowed	13 (3.8)	2 (2.0)	17 (1.8)
Divorced	16 (4.7)	5 (5.1)	100 (10.4)
Family history			
Yes	74 (21.3)*	31 (31.6)	261 (27.3)
Sex			
Male	153 (43.6)	44 (44.9)	516 (53.4)
Allergy history			
Yes	37 (10.5)	16 (16.3)	78 (8.1)*
Educational attainment			
Primary school or below	136 (40.4)*	18 (18.4)	458 (49.5)*
Junior middle or vocational school	93 (27.6)	32 (32.7)	236 (25.5)
Senior high or junior college	66 (19.6)	34 (34.7)	144 (15.6)
University and above	42 (12.5)	14 (14.3)	88 (9.5)
Trigger events			
Yes	188 (53.9)	48 (49.0)	289 (29.9)*
Ethnicity			
Han	341 (98)	94 (97.9)	932 (97.8)
Minority	7 (2)	2 (2.1)	21 (2.2)
Season of conception			
Spring	84 (24.0)	27 (27.6)	241 (24.9)
Summer	90 (25.7)	25 (25.5)	278 (28.7)
Autumn	86 (24.6)	16 (16.3)	209 (21.6)
Winter	90 (25.7)	30 (30.6)	239 (24.1)
Onset age			
Median (IQR)	36 (22–52)	34 (21–49)	24 (19–31)^*^

*IQR* interquartile range, *PMD* psychotic major depression, *NMD* non-psychotic major depression, *SZ* schizophrenia

^*^P < 0.05 compared with PMD patients

Patients with PMD were significantly more likely to have experienced trigger events within 1 year before the first episode of psychosis (*χ*^2^ = 14.939, *p* < 0.001) compared with the SZ group. No significant difference in trigger events were found between the PMD and NMD groups (*χ*^2^ = 0.734, *p* = 0.379). Compared with the NMD group, the PMD group was significantly more likely to have a family history of psychiatric diseases (*χ*^2^ = 4.567, *p* = 0.033), but this was not found between the PMD and SZ groups (*χ*^2^ = 0.833, *p* = 0.362). There were fewer patients conceived in autumn in the PMD group (16.3%, *n* = 6) than in the NMD (24.6%, *n* = 86) and SZ (21.6%, *n* = 209) groups. However, these differences were not statistically significant. In addition, there were no significant differences between the groups for sex or ethnicity.

### Missing data

Table 2 shows the comparison of missing data between the PMD, SZ, and NMD groups. There were no significant differences between the diagnostic groups in terms of missing data.

**Table 2:** Distribution of missing data

Diagnosis	NMD	PMD	SZ	*Χ*^2^	*P*
Sex	0/351	0/98	0/967		
Ethnicity	3/351	2/98	14/967	1.296	0.493
Marital status	7/351	0/98	8/967	3.496	0.174
Allergy history	0/351	0/98	0/967		
Trigger events	2/351	0/98	1/967	2.724	0.335
Educational attainment	14/351	0/98	41/967	5.080	0.080
Age at onset	2/349	0/98	21/967	5.148	0.058
Season of conception	0/351	0/98	0/967		

#### PMD vs NMD

Table 3 shows the adjusted ORs for the associations between each risk factor and diagnostic groups using the NMD group as the reference group. Having a family history of psychiatric disease was significantly associated with a diagnosis of PMD (*p* = 0.039, OR 1.701, 95% confidence interval [CI] 1.019–2.804). For every additional educational level, the risk of psychotic symptoms increased by 1.451 (*p* < 0.001, OR 1.451, 95% CI 1.168–1.808), with the NMD group as the reference.

**Table 3:** Odds ratios and 95% CIs compared PMD with NMD, adjusted for sex and family history

Parameter	*B*	Std. error	*Z*	*df*	*P*		OR	95% CI for OR	
								Lower	Upper
Intercept	− 2.189	0.315	48.233	1		< 0.001	0.112	0.059	0.205
Educational attainment	0.372	0.111	11.169	1		** < **0.001	1.451	1.168	1.808
Sex									
Male	0^a^						1		
Female	− 0.110	0.235	0.218	1		0.640	0.896	0.566	1.423
Family history									
No	0^a^						1		
Yes	0.531	0.258	4.252	1		0.039	1.701	1.019	2.804

#### Schizophrenia vs PMD

Results of the logistic analysis comparing schizophrenia with PMD (reference) are presented in Table 4. Education (*p* < 0.001, OR 0.604, 95% CI 0.492–0.741), having an allergy history (*p* = 0.01, OR 0.433, 95% CI 0.233–0.842), presence of trigger events (*p* < 0.001, OR 0.420, 95% CI 0.267–0.661), and age at first onset (*p* < 0.001, OR 0.934, 95% CI 0.914–0.954) significantly differentiated the diagnostic groups when family history and sex were controlled in the model. Being divorced (*p* = 0.037) remained significant (OR 3.087, 95% CI 1.168–10.196) compared schizophrenia to PMD.

**Table 4:** Odds ratios and 95% CIs compared schizophrenia with PMD, adjusted for sex and family history

Parameter	*B*	Std. error	*Z*	*df*		*P*	OR	95% CI for OR	
								Lower	Upper
Intercept	5.732	0.435	173.765		1	0	308.497	135.634	748.346
Educational attainment	− 0.504	0.104	23.358		1	< 0.001	0.604	0.492	0.741
Allergy history									
No	0^a^						1		
Yes	− 0.838	0.326	6.605		1	0.010	0.433	0.233	0.842
Trigger events									
No	0^a^						1		
Yes	− 0.867	0.231	14.130		1	< 0.001	0.420	0.267	0.661
Marital status									
Unmarried	0^a^						1		
Married	0.231	0.305	0.575		1	0.448	1.260	0.695	2.303
Widowed	0.980	0.874	1.257		1	0.262	2.665	0.567	19.959
Divorced	1.127	0.541	4.339		1	0.037	3.087	1.168	10.096
Family history									
No	0^a^						1		
Yes	− 0.378	0.249	2.313		1	0.128	0.685	0.424	1.126
Age at first onset	− 0.068	− 0.011	39.791		1	** < **0.001	0.934	0.914	0.954
Sex									
Male	0^a^						1		
Female	0.071	0.244	0.087		1	0.768	1.074	0.667	1.736

## Discussion

As expected, our findings indicate that PMD is more like NMD than SZ. PMD patients differed from NMD patients only in educational level and family history of psychiatric diseases. Older age of onset, presence of trigger events during the year before the first episode, not being divorced, higher level of education, and having a history of allergy helped to differentiate PMD from SZ. The results were generally in line with previous studies.

People with PMD were more likely to have a family history of a psychiatric diseases compared with people with NMD, which is consistent with a previous study [18]. To our surprise, we found no difference in family history of psychiatric diseases between the PMD and SZ groups. This highlights a potential link between heredity and psychotic symptoms.

Our findings concerning age of onset in the PMD and SZ groups are consistent with earlier studies [19, 22]. Previous studies that compared age of onset between PMD and NMD patients were inconsistent. In some studies, patients with PMD had a younger age of onset [22], whereas in others, there was no significant difference [37]. In our study, the age at first episode did not differ between PMD and NMD.

A previous study compared PMD and schizophrenia patients with a population-based sample of controls without a history of psychosis and showed that PMD and SZ patients experienced a major life event in the year before onset more often than controls, though the difference did not reach statistical significance (*p* = 0.058 for PMD and *p* = 0.056 for SZ) [17]. In the present study, more patients with PMD reported stressful life events within the year before onset compared with schizophrenia patients, which suggests a difference in the pathological mechanisms between the two disorders. However, it is important to note that patients with SZ may be more likely to underreport trigger events because of their difficulties in cooperating with doctors [38].

To our knowledge, this is the first study that included allergy history in the analysis. Surprisingly, we found a significant difference between patients with SZ with PMD. Our findings revealed that PMD patients were more likely to have had an allergy compared with SZ patients. Several lines of evidence have identified a link between non-celiac gluten sensitivity and symptoms of neuropsychiatric disorders [39, 40]. In our study, we included history of all types of allergies (e.g., drug, food, and pollen allergies). Although this result requires further verification, it does offer some insight for pathological studies focused on PMD.

For sociodemographic factors, season of conception was not different between any of the groups. We also found that the higher the level of education, the more likely the patients experienced psychotic symptoms, which is inconsistent with previous studies that reported no difference or a reverse relationship in patients with NMD and PMD [4, 19, 41]. These inconsistencies may be attributed to these studies defining education based on possession of a college degree or not [4, 41], whereas our study divided education into four levels.

We found no significant sex differences between the diagnostic groups. Differences in sample size may contribute to the discrepant findings between our and previous studies [19, 22].

As expected, having been divorced had a strong effect for identifying SZ from PMD in the current study, which is in accordance with a previous study [23].

Differences between Chinese ethnic minority and Han populations have received little attention in previous epidemiological studies that compared PMD patients with NMD and SZ patients. Our finding that ethnicity did not differ between groups was not surprising because there were very few patients who belonged to the ethnic minority group.

## Limitations

There are some limitations in this study. For example, because of the small sample size, the effects of some factors (such as ethnicity and marital status) could not be fully tested. Furthermore, patient data were retrieved from medical records, which sometimes lack information, and thus may lead to some bias. Additionally, presence of trigger events was determined based on patients’ self-report and not using a validated tool. Structured clinical interview using DSM criteria, or research diagnostic criteria would have significantly strengthened the validity of this study.

## Conclusions

Our results suggest that patients with PMD are similar to NMD patients in terms of demographic and clinical characteristics. PMD patients were more likely to have a family history of psychiatric illness in their first-, second-, or third-degree relatives and a higher level of education. We found that there were more differences between patients with PMD and those with SZ. These differences may be due to differing pathological mechanisms underlying each condition.

Season of conception did not differentiate PMD from NMD or SZ, while allergy history should be considered in future epidemiological studies of psychotic disorders. The relationship between family history and psychotic features needs further exploration. Further work on the differential etiology of PMD from other psychoses is needed.

## Supplementary Information

**Additional file 1.** Data

## Data Availability

Research data have been uploaded with the paper in Additional file 1 “data.xlsx” and can be requested from doramaia@sina.com.

## Abbreviations

PMD: Psychotic major depression
NMD: Non-psychotic major depression
SZ: Schizophrenia
OR: Odds ratios
CI: Confidence interval
